# Myxospermy Evolution in Brassicaceae: A Highly Complex and Diverse Trait with *Arabidopsis* as an Uncommon Model

**DOI:** 10.3390/cells10092470

**Published:** 2021-09-18

**Authors:** Sébastien Viudes, Christophe Dunand, Vincent Burlat

**Affiliations:** Laboratoire de Recherche en Sciences Végétales, Université de Toulouse, CNRS, UPS, Toulouse INP, 31320 Auzeville-Tolosane, France; sebastien.viudes@lrsv.ups-tlse.fr

**Keywords:** *Arabidopsis thaliana* MSC toolbox gene orthologs, Brassicaceae species, cell wall microdomains, diversity, evolution, mucilage secretory cells (MSCs), seed mucilage (SM)

## Abstract

The ability to extrude mucilage upon seed imbibition (myxospermy) occurs in several Angiosperm taxonomic groups, but its ancestral nature or evolutionary convergence origin remains misunderstood. We investigated seed mucilage evolution in the Brassicaceae family with comparison to the knowledge accumulated in *Arabidopsis thaliana*. The myxospermy occurrence was evaluated in 27 Brassicaceae species. Phenotyping included mucilage secretory cell morphology and topochemistry to highlight subtle myxospermy traits. In parallel, computational biology was driven on the one hundred genes constituting the so-called *A. thaliana* mucilage secretory cell toolbox to confront their sequence conservation to the observed phenotypes. Mucilage secretory cells show high morphology diversity; the three studied *Arabidopsis* species had a specific extrusion modality compared to the other studied Brassicaceae species. Orthologous genes from the *A. thaliana* mucilage secretory cell toolbox were mostly found in all studied species without correlation with the occurrence of myxospermy or even more sub-cellular traits. Seed mucilage may be an ancestral feature of the Brassicaceae family. It consists of highly diverse subtle traits, probably underlined by several genes not yet characterized in *A. thaliana* or by species-specific genes. Therefore, *A. thaliana* is probably not a sufficient reference for future myxospermy evo–devo studies.

## 1. Introduction

During land plant evolution, secondary growth and seeds were major innovations leading to the impressive species radiation currently observed in spermatophytes (seed plants) [1]. The adaptive capacity of seeds comes from their layered compartments surrounding the embryo with endosperm and seed coat, providing nutrients and protection, respectively, with both participating to seed physiology and developmental regulation [2]. The seed coat is a maternal tissue originating from the ovule integument that shows a high variability in architecture and thickness among seed plants [3]. In *Arabidopsis thaliana*, five outer cell layers undergo specialization during seed development, to finally be dehydrated and form a compacted seed coat in the mature dry seed. In this dead tissue, the two main layers are the inner integument enriched in pigments and the outer integument accumulating polysaccharides [4]. In *A. thaliana*, the outermost cell layer of the outer integument (oi2), also called mucilage secretory cells (MSCs), is composed of polarized epidermal cells, with a volcano-shaped central secondary cell wall structure called columella, surrounded by a hexagonal prominent primary cell wall viewed from the top of seed surface [5,6]. The dehydrated mucilage is compacted between the columella and the outer periclinal and radial primary cell walls [7]. Upon imbibition, the polysaccharidic mucilage swelling induces pressure towards the primary wall that simultaneously breaks at the outer periclinal/radial primary wall interface constituted by a prefragilized cell wall microdomain [8], allowing for mucilage extrusion to take place all around the seed [9]. The released seed mucilage (SM) is composed of two main layers: the adherent mucilage (AM) strongly attached to the seed coat, and the water soluble non-adherent mucilage (NAM) [10]. The AM remains attached to the top of the columella, which is also the attachment point of cellulose microfibrils that provide an AM structure skeleton [11]. *A. thaliana* SM represents 3% of seed dry mass and is mainly composed of sparsely branched rhamnogalacturonan I (RG-I) pectins [6,12]. This composition allows the use of ruthenium red to easily assess SM occurrence, and immunocytolabeling with cell-wall-specific probes for more accurate SM phenotyping [13,14]. There are about 100 known MSC toolbox genes that contain all molecular actors known to be involved in *A. thaliana* SM synthesis, regulation and release [7,15,16] (Table S1). One third of them are involved in the transcriptional regulatory pathway, half are direct actors of mucilage synthesis, secretion, modification and structure, and the remaining part regroups genes implicated in cell wall dynamics and hormone synthesis and perception (Table S1) [7].

In Angiosperms, several so-called myxospermous species spread among a majority of orders were reported to release SM with highly diverse compositions and contrasted MSC morphology [15,17]. Great variation in mucilage amount and/or compositions also occurs at the genus level, such as in the *Arabidopsis* species [18] or *Plantago* species [19,20] and even among populations, such as in *A. thaliana* [21,22] and flax (*Linum usitatissimum*) [23,24]. Those large variations make it difficult to trace back the evolutionary scenario of myxospermy only based on the global biochemical or morphological description. Thus, for inter-species comparison, the analysis of molecular actors may provide a useful additional level of information to better understand morphological changes and the myxospermy evolution. On the one hand, the highly conserved MYB-bHLH-WD40 repeat (MBW) regulatory complex in Angiosperms is positioned at the basis of the *A. thaliana* MSC toolbox transcriptional pathway. Interestingly, some actors of this complex such as TT2 and MYB5 orthologs seem to only be detected in the Rosid clade, suggesting their apparition in the common ancestor of this clade [16]. On the other hand, the molecular actors underlying intra-species variability in *A. thaliana* correspond to a few enzymes responsible for polysaccharide production and modification [18,21,25]. Beside the members of the aforementioned conserved regulatory complex, no more *A. thaliana* MSC toolbox genes were studied with such an evolutionary perspective. The aim of this article is to fulfill the remaining gap occurring between *A. thaliana* myxospermy deep knowledge and the potential ancestral myxospermous species present in the Rosid common ancestor. The Brassicaceae family constitutes an excellent taxonomic framework to initiate such a study, since it displays great MSC and SM morphological variations [18,26,27], as well as a large number of available qualitative genomic data and a well-characterized phylogeny [28,29,30].

We selected 33 Brassicaceae species with worldwide distribution that covered the phylogenetic tree. We performed a systematic phenotyping analysis of 27 out of these 33 Brassicaceae species using (i) a ruthenium red assay to evaluate the putative mucilage release, (ii) a histochemical morphological analysis of the dry seed and imbibed seed epidermis to provide insight on the morphology of MSC and of the SM release mode and (iii) an immunofluorescence analysis of selected cell wall epitopes within the MSC cell wall domains. This phenotyping analysis was compared to the *A. thaliana* MSC toolbox gene orthologs that we found while studying 32 out of these 33 Brassicaceae species. This genomic survey was completed by data mining of published seed development transcriptomic data for *A. thaliana*, *Camelina sativa*, *Brassica napus* and *Aethionema arabicum*.

## 2. Materials and Methods

### 2.1. Plant Material, Seeds and Genomes

We selected Brassicaceae species according to three criteria: (i) the species distribution among 3 lineages, containing 15 tribes, (ii) the genomic/seed transcriptomic data availability and (iii) the seed availability for phenotyping. A pool of 33 Brassicaceae species was first chosen according to the phylogenetic distribution. Seeds were available for phenotyping for 27 of them, to which we attributed a number code for the sake of clarity (Tables S2 and S3). Genomic data were available for 32 species (except species 26) and seed specific transcriptomic data were available for 4 species (species 1, 6, 13, 27) among these 27 species. Seeds were not available for 6 of the 33 species (species A to F). Two tribes were more represented than the others: the Camelineae tribe (including *A. thaliana* for fundamental research interest) and the Brassiceae tribe (including nearly all Brassicaceae domesticated crop species). Two non-Brassicaceae species, *Medicago truncatula* and *Linum usatissimum*, were added to the genome list to serve as an outgroup for phylogeny analysis. Seeds used during this study originated from commercial resources, scientific exchanges or personal harvests, as detailed in Table S2. The information on genome origin and relative quality is compiled in Table S3.

### 2.2. Dry Seed Phenotyping

The gross morphology of mature dry seed (color, size, and seed coat surface appearance) was screened for 27 species using an Epson perfection V100 photo scanner at a 6400 dpi resolution in opaque mode.

### 2.3. Phenotyping of Adherent Mucilage

In order to standardize the release and the staining of adherent SM among the 27 species, we used the ruthenium red staining protocol previously described [8] with a unique modification (seeds were first imbibed for 5 min in a 0.1% aqueous solution of calcofluor to also visualize cellulose microfibrils). The seeds were observed with a Leica DMIRB/E inverted microscope using a Leica MC190HD camera in the bright field mode and fluorescent mode using a UV filter set (excitation: 387/11 nm; dichroic mirror: 405 nm; emission: 440/40 nm) for ruthenium red and calcofluor staining, respectively.

### 2.4. Cross Section of Mucilage Secretory Cells, Histochemistry and Immunofluorescence

Dry seeds were first individually punctured with an ultrathin needle under a dissecting microscope to facilitate further fixation. Dry seeds from 27 species were fixed in a fixative solution (1.25% glutaraldehyde/2% paraformaldehyde in 0.05 M PIPES, 5 mM EGTA, 5 mM MgSO_4_, pH 6.9 buffer complemented with 0.1% Triton X-100 and 50% ethanol to avoid SM release and have a view of intact epidermal cells/MSCs. Further infiltration with LRW resin used a EM AMW (Automatic Microwave Tissue Processor for Electron Microscopy, Leica Microsystèmes SAS, Nanterre, France), as previously described [8]. Imbibed seeds were similarly processed to study the SM releasing mode, except that ethanol was removed from the fixative solution. Semi-thin cross sections (1 µm-thick) were cut using a histo diamond knife (Diatome) and an Ultracut E Reicher Ultramicrotome. Serial sections were spread on silane-coated slides to constitute series of species-specific slides comprising numerous serial sections, as well as two series of tissue arrays encompassing the whole panel of dry seeds or of imbibed seeds, respectively. Tissue arrays and species-specific slides were simultaneously stained for 1 min at 60 °C in 0.05% Toluidine blue in 0.1 M acetate buffer pH 4.6 for morphology. Tissue arrays were also processed for immunofluorescence as previously described [8] using selected monoclonal antibodies, allowing one to visualize the cell wall domain important for MSC opening in *A. thaliana* (LM20) [8] and the main component of the *A. thaliana* mucilage (INRA-RU2) [13], respectively. The LM20 rat monoclonal antibody specific for partially demethylesterified homogalacturonans [31,32] and the INRA-RU2 mouse monoclonal [13] specific for the RGI backbone were used at 1:10 dilution and indirectly detected using anti-rat Ig-A488 and anti-mouse Ig-A488 (Invitrogen, Thermofisher Scientific, Walthman, MA, USA), respectively. Slides were mounted under a coverslip inc prolong gold anti-fade (Invitrogen, Thermofisher Scientific, Walthman, MA, USA) and scanned at high resolution (20× or 40×) with a nano 2.0RS Hamamatsu slide scanner in the bright field mode and fluorescent mode (FITC filter set) for Toluidine blue and immunofluorescence slides, respectively. Scanned images were analyzed with NDP view (Hamamatsu), and figures were mounted using Corel Photopaint (CorelDraw graphics suite X6, Ottawa, ON, Canada), using drawings made with Microsoft PowerPoint (Microsoft office 2007, Redmond, WA, USA). Grey panels corresponded to unavailable or unexploitable samples.

### 2.5. Genomic Data Mining of A. thaliana MSC Toolbox Gene Orthologs

Coding DNA Sequences (CDS) of 32 Brassicaceae species and 2 outgroup species were used directly from available annotated genomes or predicted with fgenesh software for species without annotation (Table S3) [33]. Every CDS belonging to each Brassicaceae species was concatenated in a single multifasta file for further ortholog data mining. Each MSC toolbox protein sequence of *A. thaliana* collected from Phythozome was blasted against the concatened Brassicaceae multi-CDS file using tblastn (ncbi-blast, version 2.7.1+, https://www.ncbi.nlm.nih.gov/, (accessed on 30 August 2021)). Only the 1000 first hits, or all hits with an e-value lower than 10-10, were recovered. Each blast output was aligned using mafft (version 7.313, https://mafft.cbrc.jp/alignment/software/, (accessed on 30 August 2021)) and gap deletion was applied for a gap shared in 10% or more of the sequences using trimal (version 1.4.1, http://trimal.cgenomics.org/, (accessed on 30 August 2021)). Phylogenetic trees were built using iqtree (version 1.6.7, http://www.iqtree.org/, (accessed on 30 August 2021)) with 10,000 bootstraps from these alignments, and the clade containing the initial *A. thaliana* MSC toolbox gene up to *M. truncatula* and *L. usitatissimum* sequence(s) was extracted. The obtained tree was rooted on the branch containing these two species, because they do not belong to the Brassicaceae family and consequently constitute an outgroup for the other species. As Brassicaceae species genes are often duplicated from their common ancestor, several phylogeny trees may contain paralog gene(s) of the *A. thaliana* gene of interest and their corresponding orthologs. In such cases, only genes of the sub clade containing the *A. thaliana* MSC toolbox gene and delimited by the closest *A. arabicum* sequence, as it is the early divergent species of the studied Brassicaceaes species, were considered as orthologs.

### 2.6. Transcriptomic Data Mining of A. thaliana MSC Toolbox Gene Orthologs from A. thaliana, C. sativa, B. napus and A. arabicum Public Seed Development Transcriptomes

The whole seed development kinetic expression values were retrieved: for *A. thaliana*, 6 seed developmental stages [34], for *C. sativa*, 6 cumulated seed developmental stages [35,36], for *B. napus*, 4 seed developmental stages [37] and for *A. arabicum*, 3 developmental stages of myxospermous vs. non-myxospermous dimorphic diaspores [38]. For each gene in each species, the expression values of whole seed development kinetics samples were summed and ranked using Microsoft Excel. The mean SUM expression values for the top 10 genes in each species were used as references to calculate the relative sum expression percentage of orthologous genes of *A. thaliana* MSC toolbox genes (Table S1) in each species. For *C. sativa*, we used the previously defined correlation table [39] to identify, in the hybridized genomes, the homeologous genes corresponding to *A. thaliana* genes, and the expression values of each homeologous gene corresponding to an *A. thaliana* gene were first summed (details of homeologs are shown on the right part of Table S10). For *B. napus*, the same addition was originally made [37]. To highlight the differences in the columns “SUM%”, a common red (40)-to yellow (1)-to blue (0) heatmap was drawn using Microsoft Excel.

## 3. Results

### 3.1. Phylogenic Relationship between 27 Selected Species and Their Mature Dry Seed Whole Morphology

**Figure 1:**
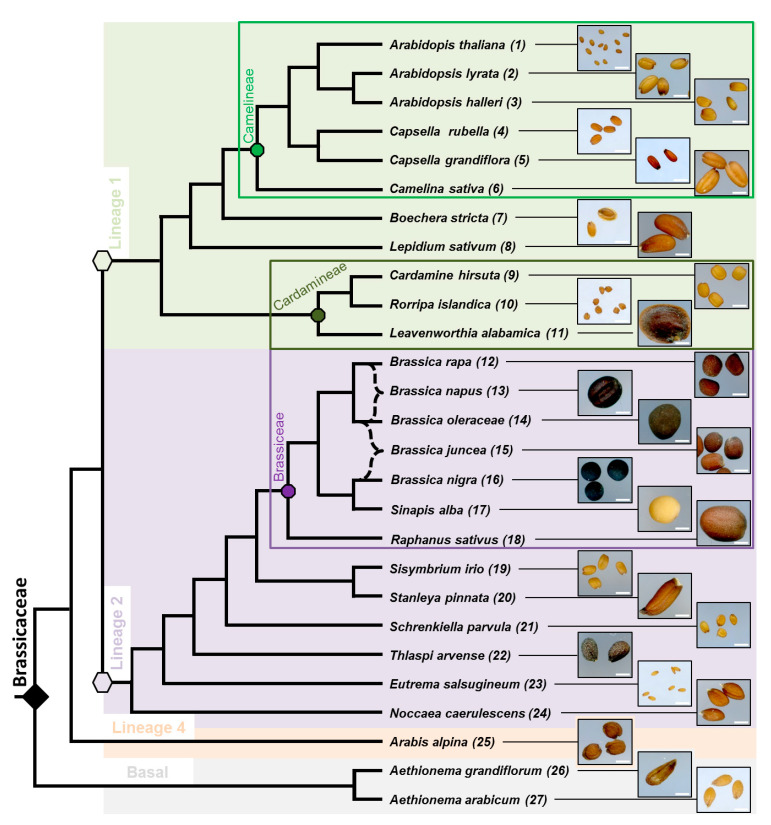
Simplified phylogenetic tree of the 27 phenotyped Brassicaceae species and illustration of their dry seed phenotype. Phylogenetic relationships between species are based on [28,29,30]. For sake of clarity, the species names were each associated with a number used to facilitate their identification along the manuscript. Lineages are highlighted by different background colors and the three tribes containing more than one species are labeled by colored rectangles. Note the high diversity of dry seed size and morphology observed with high resolution scanning. Scale bar: 1 mm.

In total, 27 Brassicaceae species were chosen in order to obtain available seeds and genomic data for 26 of them, and to provide a good taxonomic coverage among the family (Figure 1). The numbering in brackets following each species is used in all the figures and text to simplify their descriptions.

Lineage 1 (11 species) and 2 (13 species) are the most represented because they contain the well-studied tribes Camelineae (six species) and Brassiceae (seven species). Camelineae is the tribe of *A. thaliana* (1) and Brassiceae contain a lot of cultivated species, such as rapeseed (*Brassica napus* (13)). Note that the unusual branch style used for *B. napus* (13) and *Brassica juncea* (15) symbolizes their peculiar origins. Both species evolved from a common ancestor coming from the hybridization between the two connected species indicated within the tree (Figure 1) to form the current species. Within the family, seed color, size and shape show wide variation between species. All Camelineae seeds are oblong with different sizes (from 0.5 to 5 mm). Among the family, some seeds belonging to different tribes are flattened and winged, such as in *Boechera stricta* (7), *Leavenworthia alabamica* (11) and *Arabis alpina* (25). Then, these morphological modifications are probably due to evolutionary convergence of the two related traits in the Brassicaceae. Spherical seeds are only found in the Brassiceae tribe despite their contrasted colors, as exemplified by the black and white mustards (*Brassica nigra* (16) and *Sinapis alba* (17)). Then, this seed shape change should have occurred in the common ancestor of the Brassiceae. *A. arabicum* (27) has dimorphic seeds [40]; only the myxospermous morphotype is shown here.

### 3.2. Myxospermy Occurrence and Morphology at the Whole Seed Level

**Figure 2:**
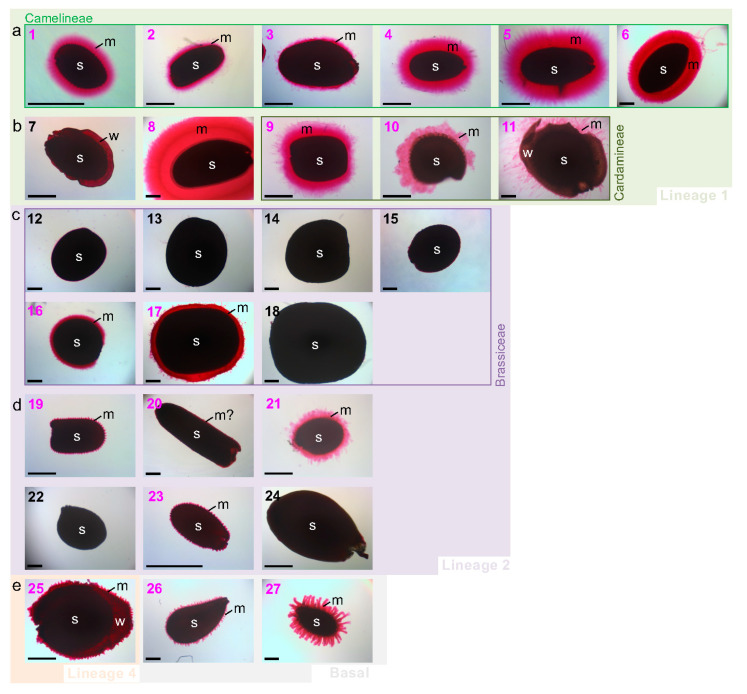
Among the 27 Brassicaceae species studied, 19 appear as myxospermous and 8 as non-myxospermous, regardless of their phylogenetic position. Camelineae lineage 1 species (**a**), other lineage 1 species (**b**), Brassiceae lineage 2 species (**c**), other lineage 2 species (**d**), lineage 4 and basal species (**e**). Following a strong shaking of imbibed seeds to facilitate the extrusion of putative mucilage, the adherent pectinaceous mucilage was stained in pink by ruthenium red in a standardized manner among the 27 species. Species numbers presented in Figure 1 were used to identify the species and colored in pink and black for the 19 myxospermous and 8 non-myxospermous species, respectively. Most of myxospermous species display a clearly visible polymorphic pink mucilage halo, while four myxospermous species (19, 20, 23, 26) show a faint mucilage halo (see Figure S1 for additional view) as compared to the non-myxospermous species (7, 12–15, 18, 22, 24). Note that the polarized pink staining in *B. stricta* (7) corresponds to staining of the pectinaceous cell wall material from the thin wing allowing light transmission. The color background, image framing and image spaces reflects the phylogenetic distribution of the species (see Figure 1 for more detail). Scale bar: 500 µm.

Within Camelineae, three *Arabidopsis* species (1–3), two *Capsella* species (4, 5) and *Camelina sativa* (6) extruded a ruthenium red-stained pectinaceous adherent mucilage, (AM) similarly to *A. thaliana* (1) used as a control (Figure 2).

Despite a common cohesion with the seed surface, the thickness and the flatness of the AM periphery can change between species. The other species of lineage 1 have cohesive AM similar to Camelineae, such as *Lepidium sativum* (8), *Cardamine hirsuta* (9), and *Rorripa islandica* (10) (Figure 2). However, *Boechera stricta* (7) does not extrude AM (the faint pink ruthenium red staining corresponds to light transmission through the thin cell wall pectinaceous material of the wing rather than AM extrusion per se), and *Leavenworthia alabamica* (11) shows extruded filamentous mucilage structures (Figure 2). Interestingly, these two exceptions also correspond to the only flattened and winged seed in the lineage 1 studied species (Figure 1). In lineage 2, most studied Brassiceae species do not release mucilage (Figure 2) except for the two mustards, *B. nigra* (16) and *S. alba* (17). These two species have a cohesive layer surrounding the seed, as in Camelineae. However, they do not seem to have cellulosic content in their mucilage, contrary to all the other myxospermous species studied (Figure S1). The mucilage trait of the other studied species belonging to lineage 2 is highly diverse (Figure 2). Some do not display any AM, such as *Thlaspi arvense* (22) and *Noccaea caerulescens* (24), or show a very thin layer of AM that requires a higher resolution to be observed, such as for *Stanleya pinnata* (20). Other species of lineage 2 show the extrusion of several conical structures at the seed surface, such as *Sisymbrium irio* (19) and *Eutrema salsugineum* (23), or display large pieces of poorly cohesive mucilage for *Schrenkiela parvula* (21) (Figure 2). Finally, in the species of lineage 4, *Arabis alpina* (25) and the two early divergent species studied *Aethionema grandiflorum* (26) and *Aethionema arabicum* (27) show the extrusion of independent mucilage structures (Figure 2), similar but longer to those observed in *Sisymbrium irio* (19) and *Eutrema salsugineum* (23) (Figure 2). These structures mostly look like spines all around the seed and, in *A. alpina* (25), these occur in both the spherical part of the seed and the wing. *A. arabicum* (27) displays the longest and thickest ones that ended by a spherical shape (Figure 2).

### 3.3. Dry Seed Epidermis/MSC Morphology and MSC Opening Mode in Imbibed Seeds

**Figure 3:**
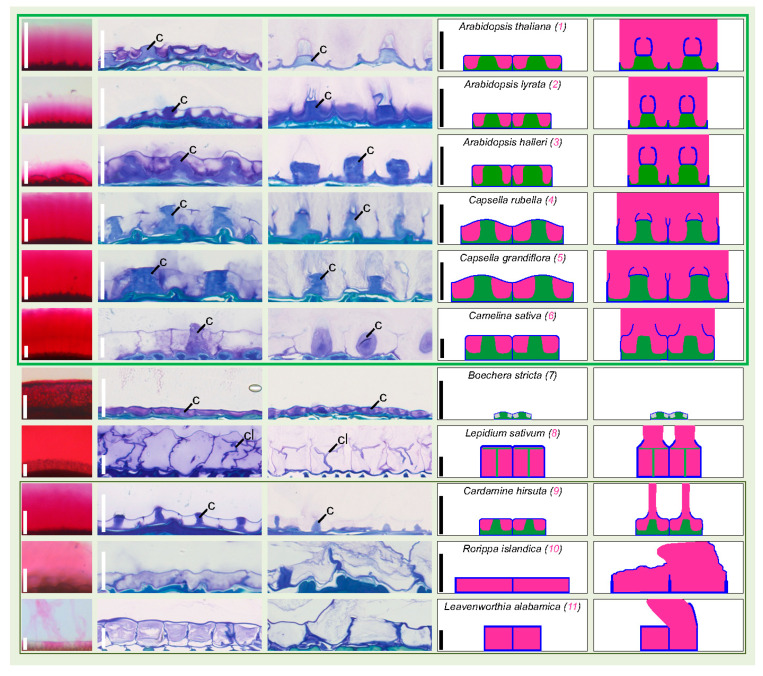
Lineage 1 dry seed MSC morphology is similar to the well-studied *A. thaliana* MSC morphology in all studied Camelineae species and highly diverse in non-Camelinae species, while the *A. thaliana* MSC opening mode is restricted to *Arabidopsis* species. Column 1: magnified view of imbibed seeds stained with ruthenium red (full images are shown in Figure 2) reminds the ability to release mucilage or not. Scale bar: 100 µm. Columns 2 and 3: Toluidine blue histochemical staining of dry seeds and imbibed seeds, respectively, focusing on the outermost cell layer morphology and of mucilage release mode for myxospermous species. Note that the inner part of the seed is located below the imaged cells. Columella (c) and columella-like structure (cl) are labeled. Scale bar: 25 µm. Columns 4 and 5: simplified drawings of two epidermal cells at the same scaling shown in Toluidine blue images. For each line the species name, the species number (pink for myxospermous and black for non-myxospermous species), as well as the scale of cross sections is reminded in column 4. A color code is applied for mucilage (pink), primary wall (blue), columella or columella-like structure (green), intracellular unidentified structures or sublayering (grey).

**Figure 4:**
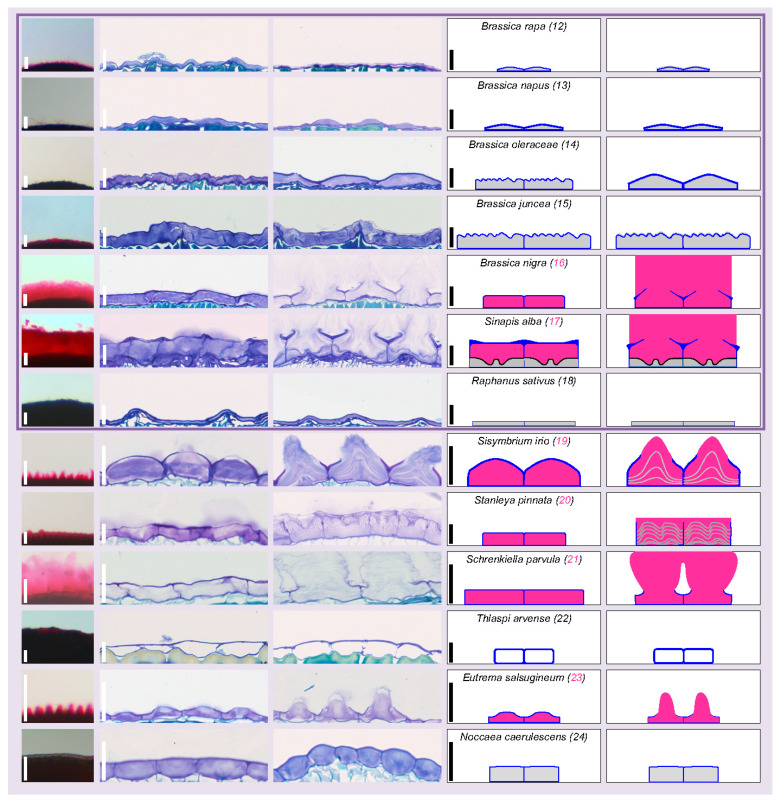
Brassiceae and other lineage 2 species seed epidermis/MSC morphology is highly diverse and contrasted as compared to lineage 1 species. Column 1: magnified view of imbibed seeds stained with ruthenium red (full images are shown in Figure 2) reminds the ability to release mucilage or not. Scale bar: 100 µm. Columns 2 and 3: Toluidine blue histochemical staining of dry seeds and imbibed seeds, respectively, focusing on the outermost cell layer morphology and of mucilage release mode for myxospermous species. Note that the inner part of the seed is located below the imaged cells. Scale bar: 25 µm. Columns 4 and 5: simplified drawings of two epidermal cells at the same scaling shown in Toluidine blue images. For each line the species name, the species number (pink for myxospermous and black for non-myxospermous species), as well as the scale of cross sections is reminded in column 4. A color code is applied for mucilage (pink), primary wall (blue), columella or columella-like structure (green), intracellular unidentified structures or sublayering (grey).

**Figure 5:**
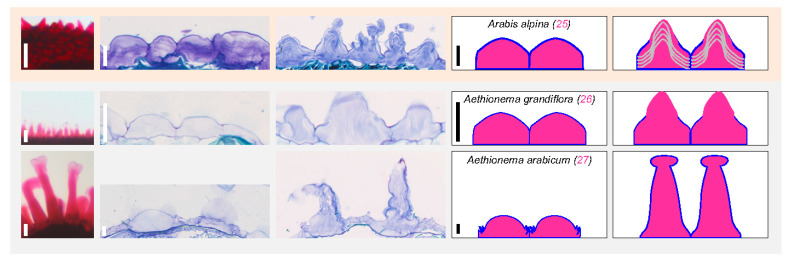
Lineage 4 and early diverging studied species display large MSCs forming a spiny-shaped mucilage upon imbibition. Column 1: magnified view of imbibed seeds stained with ruthenium red (full images are shown in Figure 2) reminds the ability to release mucilage or not. Scale bar: 100 µm. Columns 2 and 3: Toluidine blue histochemical staining of dry seeds and imbibed seeds, respectively, focusing on the MSC morphology and of mucilage release. Note that the inner part of the seed is located below the imaged cells. Scale bar: 25 µm. Columns 4 and 5: simplified drawings of two epidermal cells at the same scaling shown in Toluidine blue images. For each line the species name, the species number (pink for myxospermous species), as well as the scale of cross sections is reminded in column 4. A color code is applied for mucilage (pink), primary wall (blue), columella or columella-like structure (green), intracellular unidentified structures or sublayering (grey).

Increased resolution and additional information on the mucilage extrusion mode were obtained through histochemical study of epidermal cells before (dry seed) and after (imbibed seed) potential mucilage release, followed by simplified summary drawings for each species (Figure 3, Figure 4 and Figure 5). The metachromatic Toluidine blue O stains the primary cell wall in dark blue and the mucilage and columella in fainter blue-pinkish, whenever present. These three structures were represented with more contrasted blue, pink and green color codes, respectively, in the deduced and simplified drawings, while unidentified internal staining was represented in grey (Figure 3, Figure 4 and Figure 5). In *A. thaliana* (1) dry seed, SM is stored under the MSC primary cell wall. It forms a donut shape around the columella, a volcano-shaped secondary cell wall. Upon seed imbibition, the mucilage inflates, breaking a primary cell wall domain located at the top of radial cell walls separating each cell and allowing mucilage extrusion all around the seed, while the ruptured primary wall remains attached to the top of the columella (Figure 3). The six studied Camelineae species (1–6) display a MSC morphology very similar to *A. thaliana* before imbibition (Figure 3). They have rectangular cells delimitated by a radial cell wall with a central columella. However, after imbibition, the breaking point of the radial cell wall domain commonly observed in the three *Arabidopsis* species (1–3) seems to be different from the rupture mode observed in the two *Capsella* species (4, 5) and *C. sativa* (6) despite their phylogentic proximity (Figure 3). Contrary to the three *Arabidopsis* species, *C. sativa* has a primary wall-breaking domain localized at the top of the columella, and following imbibition, the periclinal primary CW remains attached to the radial CW. *Capsella* species show an intermediate situation between the *Arabidopsis* species and *C. sativa*, since they have a part of periclinal CW that remains attached to both the top of columella and of radial CW (Figure 3). Within lineage 1, the epidermis/MSCs of non-Camelineae species are much more diverse (Figure 3). They are all myxospermous except for *Boechera stricta* (7), which has no primary CW rupture and whose seed epidermis is also extremely reduced in size (Figure 3). An *Arabidopsis*-like columella is observed in *Boechera stricta* (7) and *Cardamine hirsuta* (9), but it can also be drastically changed in shape, as for *Lepidium sativum* (8) or simply disappear, as for *Rorippa islandica* (10) and *Leavenworthia alabamica* (11) (Figure 3). The primary CW rupture zone of *L. sativum* (8) and *C. hirsuta* (9) is central, as for *C. sativa*, while for *R. islandica* (10) and *L. alabamica* (11), the rupture occurs at the top of the radial CW as for the *Arabidopsis* species, but this only happens within a restricted number of cells (Figure 3).

Within lineage 2, the studied Brassiceae species can have more or less flattened epidermal cells, but they never possess a columella, per se, or a columella-like structure, even if *S. alba* (17) MSCs contain intracellular structures of unknown function (Figure 4). As expected with the previously described ruthenium red staining, both mustards (*B. nigra* (16) and *S. alba* (17)) are the only Brassiceae species displaying a clear extrusion with a rupture of primary CW occurring at the middle of MSC periclinal CW (Figure 4). Among the five studied species of lineage 2 that do not belong to the Brassiceae tribe, there is a great diversity of epidermal cell morphology (Figure 4). *Schrenkiella parvula* (21) MSCs are similar to mustard MSCs (no intracellular structure and central opening), but the border of released mucilage patches is more reactive to Toluidine blue (Figure 4). The released mucilage forms even more delimitated patches with spine shapes above each MSC in *Sisymbrium irio* (19) and *Eutrema salsugineum* (23), in agreement with the observed ruthenium red staining (Figure 2 and Figure 4). *Stanleya pinnata* (20) shows intracellular layered structures that increase in volume but are not extruded following imbibition, while *Noccaea caerulescens* (24) shows a similar pattern without an apparent cell volume increase (Figure 4). *Thlaspi arvense* (22) seed’s epidermis does not display particular sign of MSC differentiation, since these cells appear empty, without any change following imbibition (Figure 4).

No lineage 3 species could be observed and the only lineage 4 studied species, *Arabis alpina* (25), displays central mucilage extrusion in independent layered spine-shaped structures, similar to the lineage 2 species *S. irio* (19) and *E. salsugineum* (23) (Figure 4 and Figure 5).

Finally, the two studied early diverging species from the Brassicaceae family, *A. grandiflora* (26) and *A. arabicum* (27), release independent mucilage structures after imbibition (Figure 5). The larger size of *A. arabicum* (27) MSCs explains the larger size of the mucilage spine-shaped structure released upon imbibition. It seems that contrary to the other species releasing delimitated mucilage structure above each MSC (*A. grandiflora* (26), *A. alpina* (25), *E. salsugineum* (23), and *S. irio* (19)), *A. arabicum* (27) MSCs do not undergo CW rupture at the central position of the periclinal CW. It rather seems that the periclinal CW unfolds in an accordion during imbibition (Figure 4 and Figure 5).

### 3.4. In Situ Immunolabeling of SM Content and MSC Primary Cell Wall Microdomains

**Figure 6:**
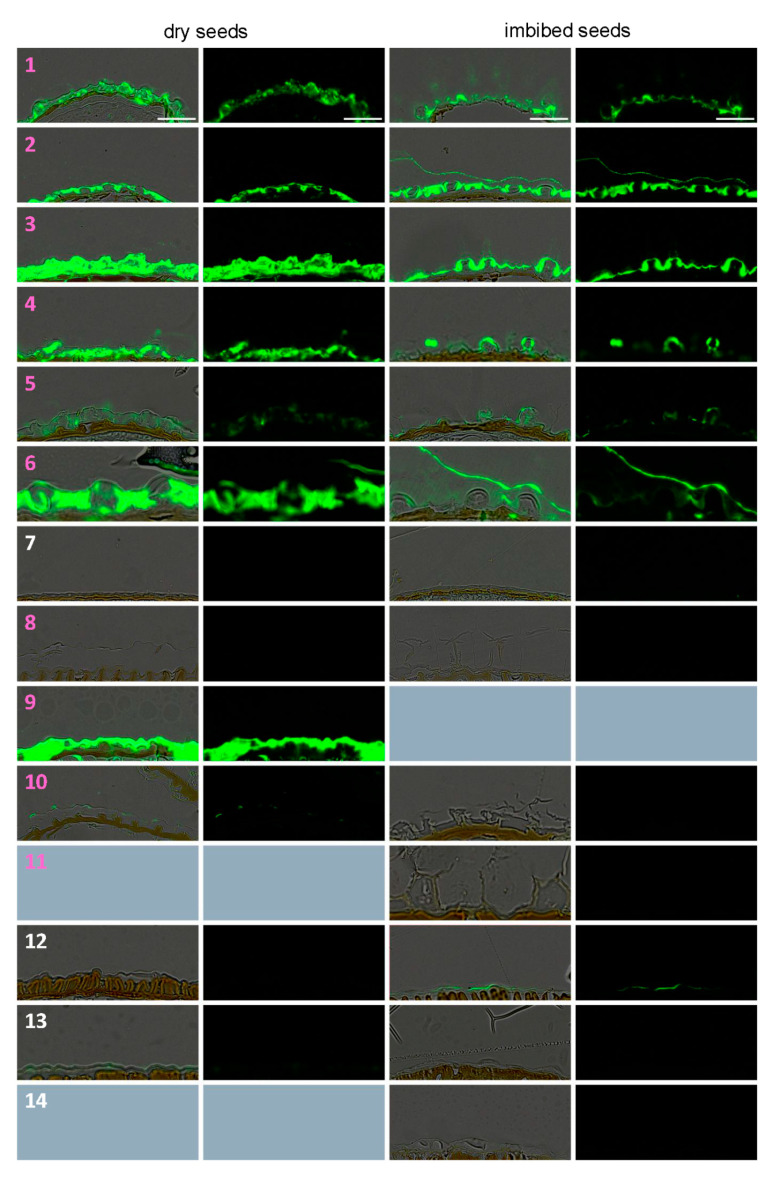

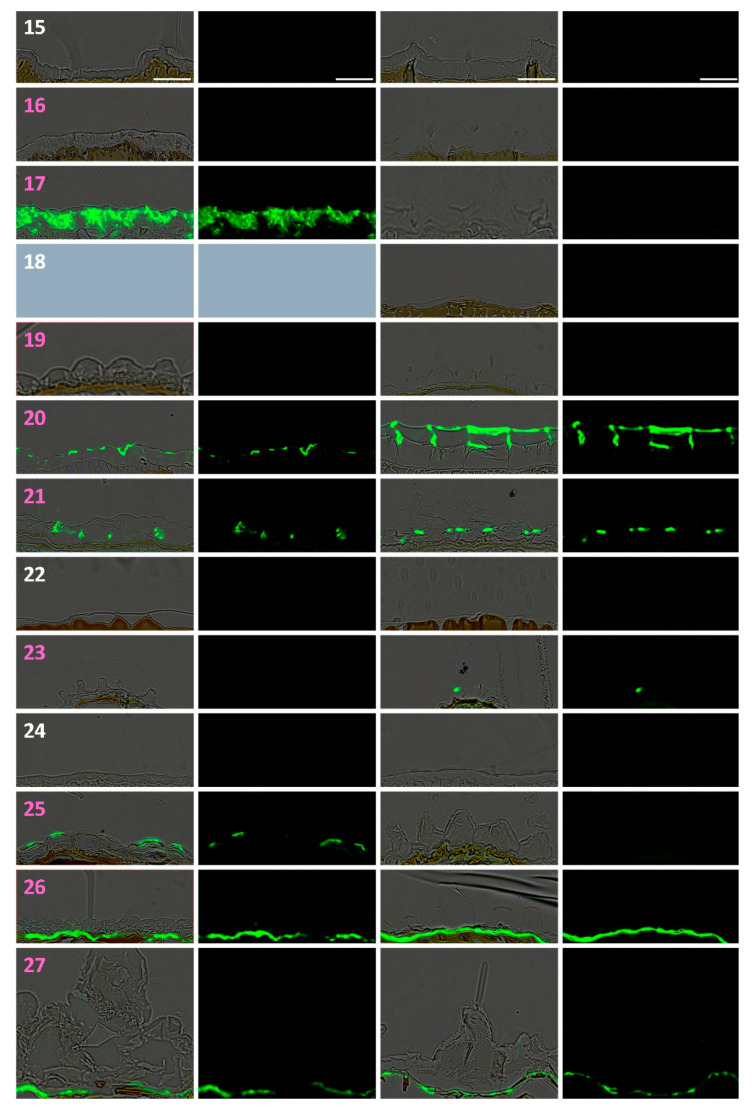
Immunofluorescence survey of mucilage in dry and imbibed seeds using the INRA-RU2 anti-RGI backbone antibody (2 pages). Serial sections of tissue arrays used for Toluidine blue staining were labeled with INRA-RU2 and anti-mouse Alexa488 as a secondary antibody. Note the recurrent occurrence of labeling in most myxospermous species regardless their position in the phylogenetic tree. Species numbers defined in Figure 1 were colored in pink and white for myxospermous and non-myxospermous species, respectively. Grey panels correspond to unavailable or unexploitable samples. Bars: 50 μm.

For some species that do not extrude mucilage, it is difficult to decipher whether the seed epidermal cells actually contain unreleased mucilage by a lack of primary CW rupture, or if these cells do not differentiate as MSCs. *A. thaliana* mucilage is enriched in sparsely branched RGI pectins labeled with INRA-RU2 monoclonal antibody [13]. RU2 can be used as a marker of mucilage presence on serial seed cross sections in parallel of sections used for Toluidine blue staining. Upon imbibition, Camelineae species maintain a strong AM signal close to the newly revealed seed surface, especially for the three *Arabidopsis* species (1–3). As expected, RU2 staining is located within MSCs of almost every myxospermous species, regardless of their position within the phylogenetic tree (Figure 6). Surprisingly, RU2 labels the cell layer just below the MSCs and not the MSCs themselves for the two *Aethionema* species (26, 27). Interestingly, *L. sativum* (8), *R. islandica* (10) and *L. alabamica* (11), which belong to lineage 1 but do not present a columella, show weak-to-absent RU2 labeling, suggesting a mucilage compositional change. *Stanleya pinata* (20) MSCs are labeled only in the upper part while they are imbibed, suggesting that they extrude mucilage and not only inflate during imbibition. *C. hirsuta* (9) probably has an enrichment of sparsely branched RGI, as the labeling of its MSC content by RU2 is the strongest in the family. The eight non-myxospermous species (*B. stricta* (7), five non-mustard Brassiceae species (12–15, 18), *Thlaspi arvense* (22), and *N. caerulescens* (24)) are not labeled with RU2, supporting the lack of pectinaceous mucilage (Figure 6).

In *A. thaliana* dry seeds, LM20 antibody labels the MSC radial CW microdomain corresponding to the rupture zone for a proper extrusion during imbibition [8] (Figure S2). Here, the studied species were screened with this antibody to evaluate two hypotheses. First, whether the epidermal cells that do not undergo rupture during imbibition lack this specific domain. Second, whether the shift in the rupture zone location from the periphery to the center could be correlated with a shift of the LM20 labeling zone. The periclinal primary CW of almost every studied myxospermous and non-myxospermous species displays a signal for LM20 (Figure S2). Only two species do not have labeling: the non-myxospermous species *R. sativus* (18) and *T. arvense* (22), which interestingly have very atypical epidermal cells, namely the thinnest but wider ones and the only empty ones, respectively. The labeling can be continuous as for *L. alabamica* (11) and all studied Brassiceae species (12–17) or discontinuous, as in *A. thaliana* (1). For a discontinuous signal it may be difficult to decipher the localization of the labeling, since the perfect cutting plan is not always available on tissue arrays. The myxospermous species with a spiny morphology of MSCs (19, 23, 25–27) show an interesting central labeling in dry seed MSCs, and from either side of the opened area following imbibition. For *S. pinnata* (20), the LM20 labeling of imbibed MSCs is located just below the labeling zone observed for RU2, highlighting again the potential extrusion of this species through the lateral zone of MSCs.

### 3.5. Bioinformatics Insight on A. thaliana MSC Toolbox Gene Orthologs in Brassicaceae Species

**Figure 7:**
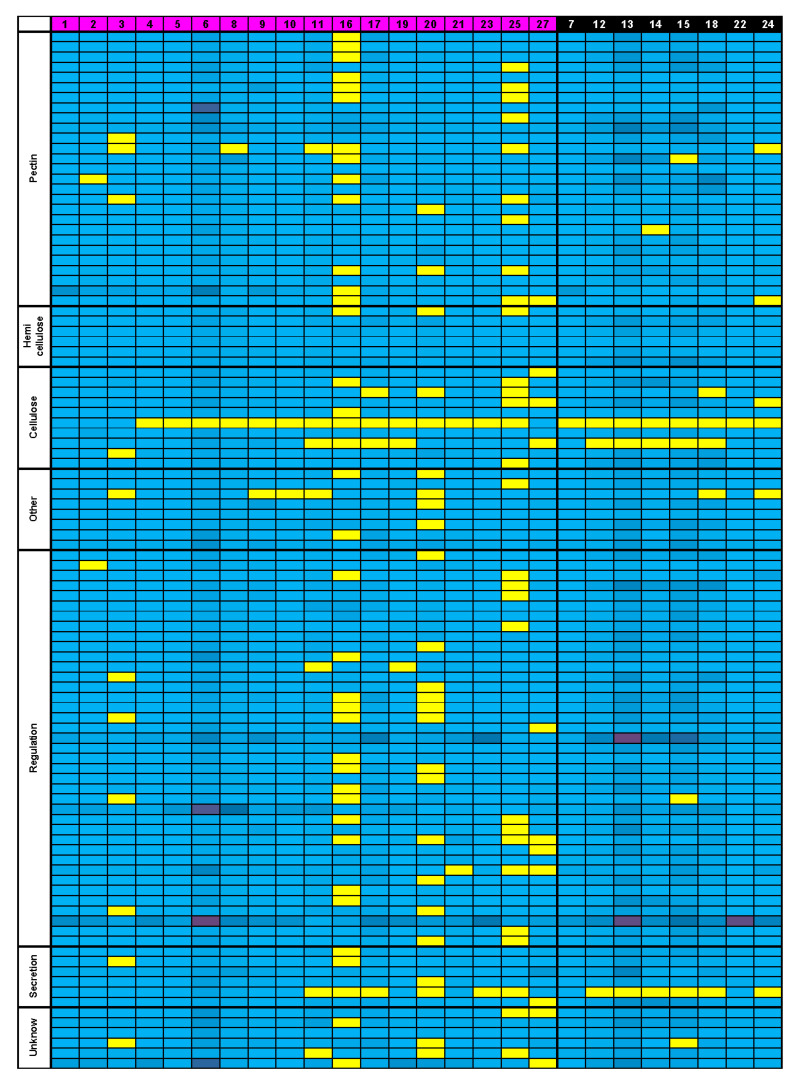
The presence of the *A. thaliana* MSC toolbox gene orthologs throughout the Brassicaceae family is unrelated to their myxospermous ability or lack thereof. Orthologous genes presence (blue) or absence (yellow) is highlighted for each *A. thaliana* MSC toolbox genes (in line) within each studied species (in column). Genes are grouped in functional categories and their numbers for each species due to duplications are symbolized by a colored gradient from light blue (1 gene) to dark purple (23 genes). Species are identified with the number attributed in Figure 1, with myxospermous ones on the left side (in pink) and non-myxospermous ones on the right (in black). See Tables S4–S9 for more details.

With the aim to highlight potential change at the genetic level between myxospermous and non-myxospermous species and to establish relationships with the subtle traits revealed by the phenotyping, we evaluated a targeted approach, identifying in 32 species, including 26 of the 27 species phenotyped in this study, the putative orthologous genes of the *A. thaliana* MSC toolbox genes characterized so far (Table S1). This approach was based on two hypotheses. First, orthologous genes of *A. thaliana* MSC toolbox genes in other Brassicaceae species may have the same functions as the ones characterized for *A. thaliana*. Second, gene(s) involved in myxospermy could have undergone pseudogenization in species that have lost myxospermy and, as a result, become dissimilar in their sequences compared to those which still have their function. Thorough data mining allowed us to find at least one orthologous gene for a large majority of *A. thaliana* MSC toolbox genes for each Brassicaceae species analyzed, including the eight studied non-myxospermous species (Figure 7, Table S4).

There are a few interesting exceptions to the general tendency of gene conservation with a single copy in each studied species. *CESA9* undergo a massive pseudogenization, as an ortholog was only found in the *Arabidopsis* species (1, 2, 3) and in the earliest diverging species, such as *A. arabicum* (27) (Table S4). *PMEI14* is an example of the rare duplication event that occurs in the common ancestor of a sub-clade of Brassicaeceae, here lineage 1, as there is more co-orthologs in it compared to the rest of the family (Table S4). This may also be the case for *DOF4.2*, *SK11-SK12*, *TBA1-TBA2-TBA3*, and *MYB75*; however, their corresponding phylogenetical trees were not sufficiently resolved to be properly interpreted. This is probably due to the short size of the sequences (Table S4). Therefore, unfortunately, no simple direct relationship could be established between the absence of *A. thaliana* MSC toolbox gene orthologs and the lack of the myxospermy trait (Figure 7). Indeed, species showing the less orthologous gene presence are myxospermous, such as *Brassica nigra* (16) and *Arabis alpina* (25), and conversely, non-myxospermous species, such as *Boechera stricta* (7), contain most of *A. thaliana* orthologs (Figure 7, Table S5). When sorting the data to look for a putative correlation between gene losses and a more subtle phenotype at the cellular level such as a columella presence (Table S6) or the position of the primary cell wall rupture zone (Table S7), no clear correspondence appears as well. In the last case, PMEI6 and PRX36 are shown to be directly linked to the fragilization of a microdomain at the top of the radial CW in *A. thaliana* [8], leading to a phenotype of defect of mucilage release, also observed in in mutant for *SBT1.7/ARA12* [41]. The presence of orthologs of these genes among species is not correlated to radial CW rupture, nor is their absence correlated to other CW rupture zones (Table S7). When grouping investigated genes by their characterized function categories, in *A. thaliana*, any decrease in the presence of orthologous genes related to mucilage polysaccharides synthesis for non-myxospermous species was observed (Table S8). As the mutations of the MSC toolbox genes do not have the same phenotypic impact on *A. thaliana*, these genes were also sorted according to their impact, but again, no enrichment of losses was found for these categories (Tables S1 and S9).

**Figure 8:**
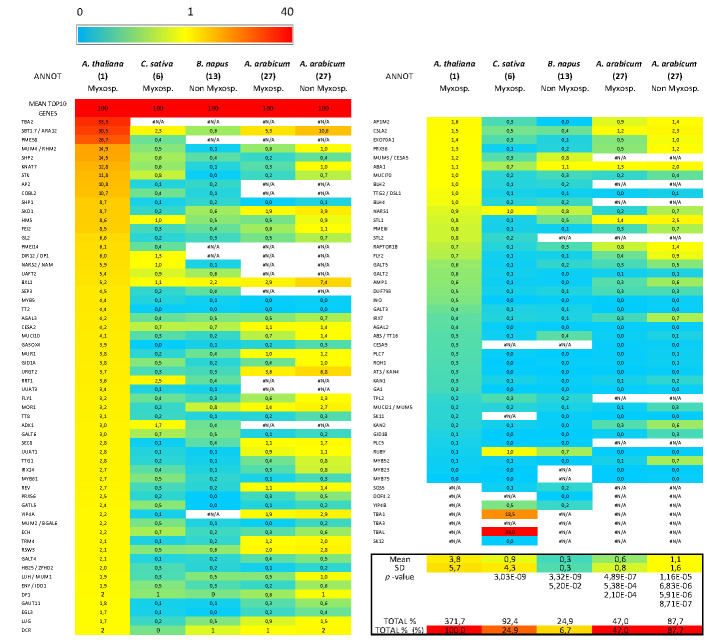
Comparison of the relative expression level of the *A. thaliana* MSC toolbox genes and of their orthologous genes from *C. sativa*, *B. napus*, and *A. arabicum* (myxospermous and non-myxospermous dimorphic diaspores) during whole seed development. The expression value of each *A. thaliana* MSC toolbox gene ortholog was summed for the whole seed development kinetics samples and were displayed in % of the similarly processed 10 most expressed genes in the whole genome for each species. Note that the relative expression values are significantly higher in *A. thaliana* as compared to the three other species. The relative expression values are slightly higher in the myxospermous species *C. sativa* than in the non-myxospermous species *B. napus* but are globally slightly higher in the non-myxospermous seeds than in the myxospermous seeds of the dimorphic species *A. arabicum*. To highlight the differences, a common red (40)-to yellow (1)-to blue (0) heatmap was used. See Table S10 and methods for detailed information and legend.

Since no clear relationship could be established between the myxospermous traits and the presence of *A. thaliana* MSC toolbox gene orthologs, we analyzed published seed development transcriptomic data from *A. thaliana*, *C. sativa*, *B. napus* and *A. arabicum*. Although these species only represent a fraction of the species targeted in this study, they had the double advantage of being spread among the Brassicaceae phylogenetic tree (Figure 1) and being either myxospermous (*A. thaliana* (1), *C. sativa* (6), *A. arabicum* (27) dehiscent diasporas) or not (*B. napus* (13), *A. arabicum* (27) non-dehiscent diasporas) (Figure 2). In an effort to unify the heterogeneity of orthologous gene expression data sources from different species, we summed the expression values from all available whole seed development kinetic samples for each gene in each species (Table S10). For each species, the summed expression values of orthologous genes of *A. thaliana* MSC toolbox genes (Table S1) were expressed as a percentage of the mean summed value from the 10 most expressed genes used as a reference (Figure 8, Table S10). Interestingly, the common top 10 genes partially occurred among species. The relative expression profile of MSC toolbox genes was clearly higher in *A. thaliana* as compared to the orthologs in other species, regardless of whether they were myxospermous or not. Despite their relatively weak expression profile, a slightly but significantly higher expression profile occurred in the myxospermous *C. sativa* as compared to the non-myxospermous *B. napus*. However, using the recent elegant transcriptomic data from the dimorphic *A. arabicum* seeds [38] (Figure 8, Table S10), the opposite trends occurred.

While considering the significant fold change ratio, 1, 22 and 14 of 105 *A. thaliana* MSC toolbox genes were upregulated in myxospermous vs. non-myxospermous seeds at 0, 1 and 30 DAP, respectively. However, 1, 21 and 17 *A. thaliana* MSC toolbox genes were conversely upregulated in non-myxospermous seeds at 0, 1 and 30 DAP, respectively [38]. Moreover, none of these upregulated MSC toolbox gene orthologs were ranked among the most differentially expressed genes [38]. Therefore, it could be speculated that the *A. thaliana* MSC toolbox genes are rather specific to the specific myxospermous sub-traits observed in *A. thaliana*, and that use of this toolbox to decipher the molecular actors of myxospermy in other species could be tricky.

## 4. Discussion

### 4.1. Is Myxospermy an Ancestral Feature of the Brassicaceae Family?

Myxospermy was found in all studied lineages, and importantly, in the two *Aethionema* species belonging to the early divergent group. This could indicate a potential presence of myxospermy in the Brassicaceae family common ancestor. In addition to the two *Aethionema* species studied here, two other *Aethionema* species were also reported to be myxospermous [26], consolidating the myxospermic ability of the early diverging clade of Brassicaceae family. Non-myxospermous species should consequently have undergone independent losses from this ancestral status. At the cellular level, several arguments can also support the hypothesis of multiple independent losses of the ancestral trait. The non-myxospermous lineage 1 species *Boechera stricta* (7) has an epidermis cell layer severely reduced in size, although these cells contain a columella and the species is a close relative to the fully myxospemous Camelineae tribe. Additionally, RU2 antibodies that label the pectinaceous mucilage of every species of lineage 1 do not label *B. stricta* epidermal cells, suggesting a loss of pectinaceous mucilage, while a biochemical change of the polysaccharide composition cannot be excluded. Altogether, it seems that *B. stricta* underwent a de-differentiation of its epidermis cell layer from MSCs to non-MSCs, avoiding the accumulation of hydrophilic polysaccharides and their extrusion, but keeping a columella whose function remains elusive. High diversity of epidermis morphology also occurs within the Brassicaea tribe. The thickest MSCs belong to the two myxospermous mustards, and the non-myxospermous species show variable epidermal cell thickness, up to the thinnest and widest cells observed in *R. sativum* (18). Contrary to *B. stricta* (7), the loss of the myxospermy trait in Brassiceae is linked to a change in epidermal cell shape. However, for Brassiceae species, it is harder to decipher whether a progressive disappearance occurred since their common ancestor, but as they are all cultivated species, it is possible that the myxospermy trait was differentially affected during the selection process, as for the pea seed coat [42]. If myxospermy can be lost through the de-differentiation of the MSC cells, the neo-functionalization of the trait could occur as well thanks to epidermis morphological change. This is the case for Cardamineae species (9–11) that have highly diverse MSC and mucilage morphology, although these are close relative species of Brassiceae, reinforcing the idea that SM can quickly evolve. This is also illustrated in lineage 2 by the highly contrasted phenotype observed between the phylogenetically relatively close myxospermous *S. pinnata* (20) and non-myxospermous *T. arvense* (22). All of these inter-species comparisons highlight how changeable myxospermy is as a trait. Moreover, *A. arabicum* (27) and *Diptychocarpus strictus* (not phenotyped here) were shown to have seeds with dimorphism on myxospermy ability [40,43]. This highlights that seed mucilage can be easily disabled or activated during seed formation without any genetic change and could be rather explained by complex dimorphism-related gene expression [38]. However, we did not observe any correlation between the global expression profile of *A. thaliana* MSC toolbox gene orthologs and dimorphism (Figure 7; Table S10) [38]. The morphological change might be correlated with subtle differential expression between the myxospermous and non-myxospermous seeds for *GL2*, *MYB61* and *SHP2* regulator genes and also more downstream actors, such as *PMEI6*, *CESA2*, *CESA5*, *CSLA2*, and *GALT5* [38]. While gene expression regulation is obviously crucial to allow or deny myxospermy establishment, large-scale analysis of coding genomic sequences from the *A. thaliana* MSC toolbox gene orthologs may not be appropriate to clear interspecies comparison. When changes in sequence are responsible for myxospermy loss, it can correspond to a deletion in a single gene, as exemplified by *MUM2* polymorphism in Shahdara *A. thaliana* natural population [44]. We had no access to such a resolution in our study but rather searched for stronger pseudogenization that should occur in non-myxospermous species for genes that have no additional function. Consequently, the absence of correlation between the myxospermy traits and the presence/absence of *A. thaliana* MSC toolbox gene orthologs in the studied Brassicaceae species could be due to the pleiotropic functions of the corresponding proteins and/or to their late co-option for mucilage traits in the Brassicaceae ancestor. However, on seven candidate genes highlighted by GWAS on flax seed mucilage content, six genes were orthologous to the *A. thaliana* MSC toolbox genes [24]. This could indicate that some of the toolbox genes can keep their function in mucilage even outside of the Brassicaceae family.

### 4.2. Arabidopsis Species as an Uncommon Model for Mucilage Secretory Cells

While some myxospermy traits detected with ruthenium red staining or RU2 immunolabeling appear widely distributed along the Brassicaceae phylogeny, other myxospermy traits are more species-specific. Among the studied myxospermous Brassicaceae species, SM extrusion occurs through the center of the MSCs, except for the three studied *Arabidopsis* species. In *A. thaliana*, the weakening of the microdomain localized at the top of radial CW leads to a proper rupture at this location upon seed imbibition [8]. Consequently, for each imbibed MSC, the piece of primary CW that cover MSCs look like an upside-down umbrella attached to the top of a columella [9]. As a columella is located in the center of MSCs, the radial opening could be a consequence of columella apparition for a proper extrusion of SM. However, *C. sativa* (6) and *C. hirsuta* (9) display a central opening as well as a columella presence. Consequently, the columella presence and rupture zone seem to be independent. However, the columella may have appeared first during evolution, as it is present in several species of lineage 1, contrary to the rupture of radial CW, which is restricted to *Arabidopsis* species. Note that for *R. islandica* (10), *L. alabamica* (11) and *S. pinnata* (20), the rupture occurs at the radial position of MSCs but as they have very distinct MSC morphology, it is probably an independent apparition. An ancestor of the Brassicaceae family should probably have MSCs without a columella with a central opening upon imbibition for mucilage extrusion that does not correspond to the *Arabidopsis* phenotype.

### 4.3. Ecological Perspective

The diversity of the observed myxospermy traits could be linked to ecological roles. The mucilage morphology of *L. alabamica* (11) and its absence in *B. stricta* (7) are interesting. Both species are the only ones of lineage 1 with flattened and winged seeds, and they show a unique mucilage phenotype and an absence of myxospermy, respectively. Both species can indicate the negative effect of cohesive mucilage on wind dispersion for winged seeds. *Dyptiocarpus strictus*, a Brassicaceae species not included in our study, confirms this incompatibility with a seed dimorphism [43]. One morphotype has cohesive mucilage around the seed but a reduced wing, while the other lacks mucilage but has a large wing. *L. alabamica* (11) mucilage could be studied for its physical influence on the seed adherence and floatability because its filamentous shape can be linked to the co-occurrence of wind and another kind of dissemination avoiding staying glued or sinking too fast.

Altogether, the present study documents the high diversity of myxospermy traits and shows the limits of using *A. thaliana* as the unique reference for myxospermy evodevo studies. This might be overcome by considering the increasing number of genomic and transcriptomic studies targeted on non-model species that could bring some of these to the status of model species.

## Data Availability

Not applicable.

## Supplementary Materials

The following are available online at https://www.mdpi.com/article/10.3390/cells10092470/s1, Figure S1: Ruthenium red and calcofluor double staining for the 19 myxospermous species highlights the cellulosic part contained in seed mucilage, Figure S2: Immunofluorescence survey of the putative occurrence of a topochemical signature of the cell wall domain rupture zone in dry and imbibed seeds using the LM20 antibody, Table S1: *A. thaliana* MSC toolbox genes, Table S2: List and seed provider of the 27 species phenotyped in this study, Table S3: List and Genomics data origin of the 32 species used in this study, Table S4: Detailed genomic data mining of *A. thaliana* MSC toolbox gene orthologs among Brassicaceae species, Table S5: Sorting of the studied species according to the myxospermy occurrence highlights the absence of correlation with the presence of *A. thaliana* MSC tool box gene orthologs related to mucilage synthesis in *Arabidopsis*, Table S6: Sorting of the studied species according to the columella occurrence highlights the absence of correlation with the presence of any *A. thaliana* MSC tool box gene orthologs, Table S7: Sorting of the studied species according to their opening zone of the primary cell wall that allows the mucilage extrusion highlights the absence of correlation with orthologs from the genes controlling the opening mode in *A. thaliana*, Table S8: Sorting of studied genes according to their polysaccharide-related functions highlights the absence of correlation with the occurrence of myxospermy, Table S9: Sorting of studied genes according to their impact in myxospermy (strength of the phenotype in the corresponding mutant) highlights the absence of correlation with the occurrence of myxospermy, Table S10 (Related to Figure 8): Comparison of the relative expression values of *A. thaliana* MSC tool box gene orthologs between *A. thaliana*, *C. sativa*, *B. napus* and *A. arabicum* whole seed development illustrates their higher relative expression values in *A. thaliana*.
