# Supplementary material for: Myxospermy Evolution in Brassicaceae: A Highly Complex and Diverse Trait with Arabidopsis as an Uncommon Model
Source: Cells. 2021 Sep 18;10(9):2470. doi: 10.3390/cells10092470 (PMC8469493; doi:10.3390/cells10092470)
Supplement: Supplementary file 1 [file cells-10-02470-s001.zip › Viudes et al_Figure S1.pdf]

Viudes et al Fig. S1 (2 pages; legend on page 2)

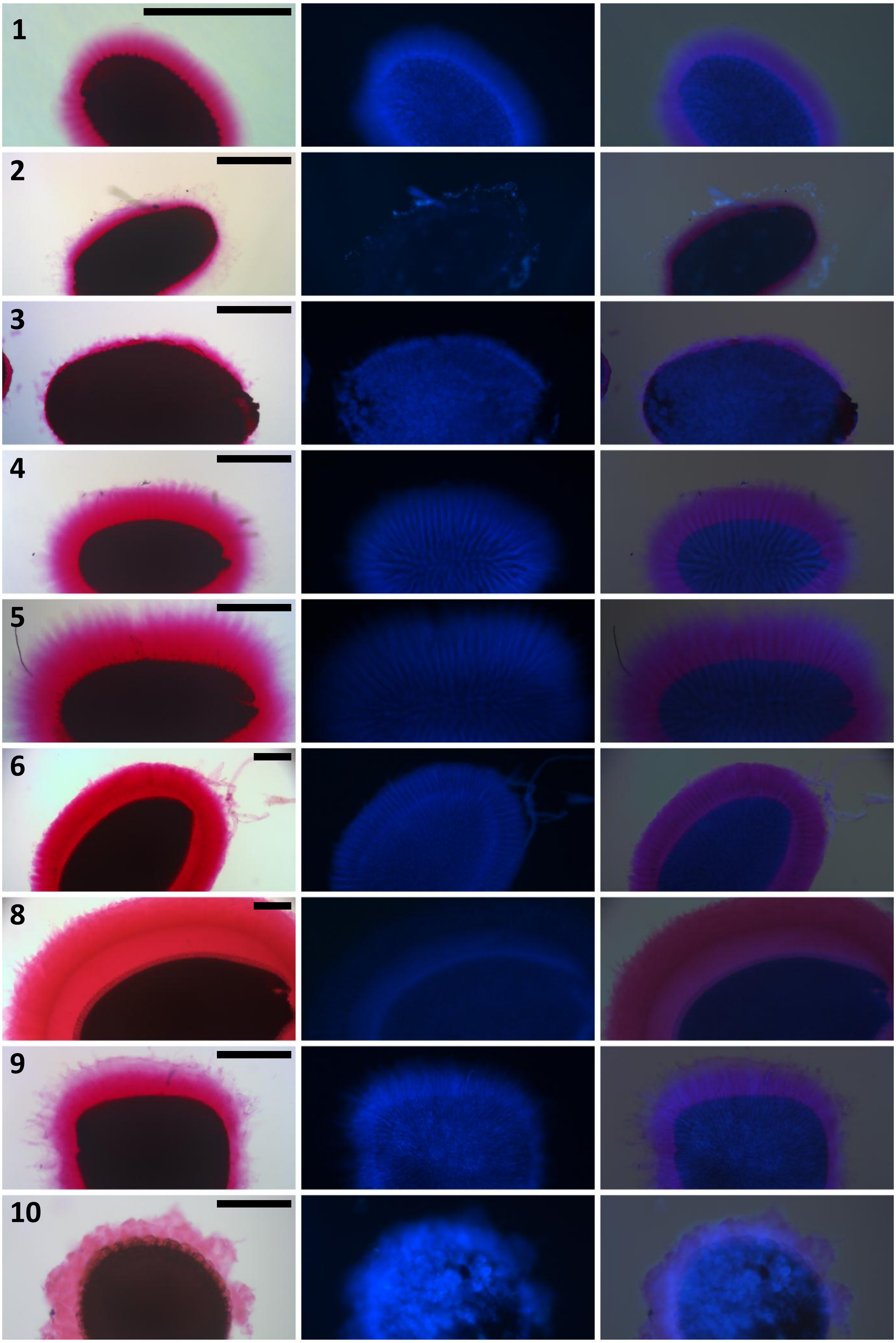

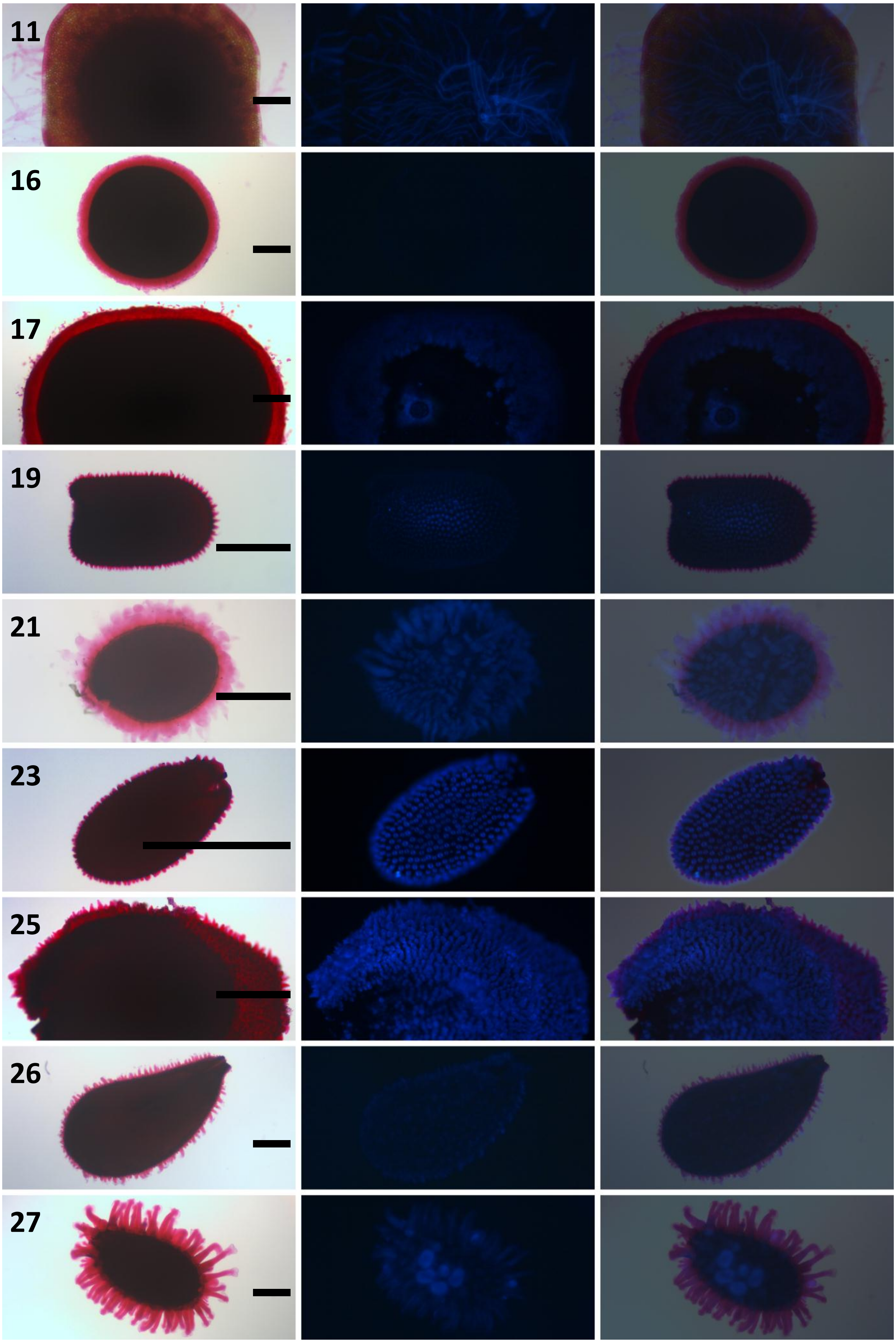

Figure S1: Ruthenium red and calcofluor double staining for the 19 myxospermous species highlights the cellulosic part contained in seed mucilage. Species numbers presented in Fig. **1** were used to label the images (only the myxospermous species are shown here). Column 1 images correspond to the ruthenium red staining after vigorous shaking shown in Fig. **2**. Column 2 shows the same seed under UV light to reveal the calcofluor labelling which is specific to cellulose. Column 3 is the merge of column 1 and 2 with a transparency set up at 70% for black background images. Bars: 500 µm.
