# Supplementary material for: Myxospermy Evolution in Brassicaceae: A Highly Complex and Diverse Trait with Arabidopsis as an Uncommon Model
Source: Cells. 2021 Sep 18;10(9):2470. doi: 10.3390/cells10092470 (PMC8469493; doi:10.3390/cells10092470)
Supplement: Supplementary file 1 [file cells-10-02470-s001.zip › Viudes et al_Figure S2.pdf]

Viudes et al Fig. S2 (2 pages; legend on page 2)

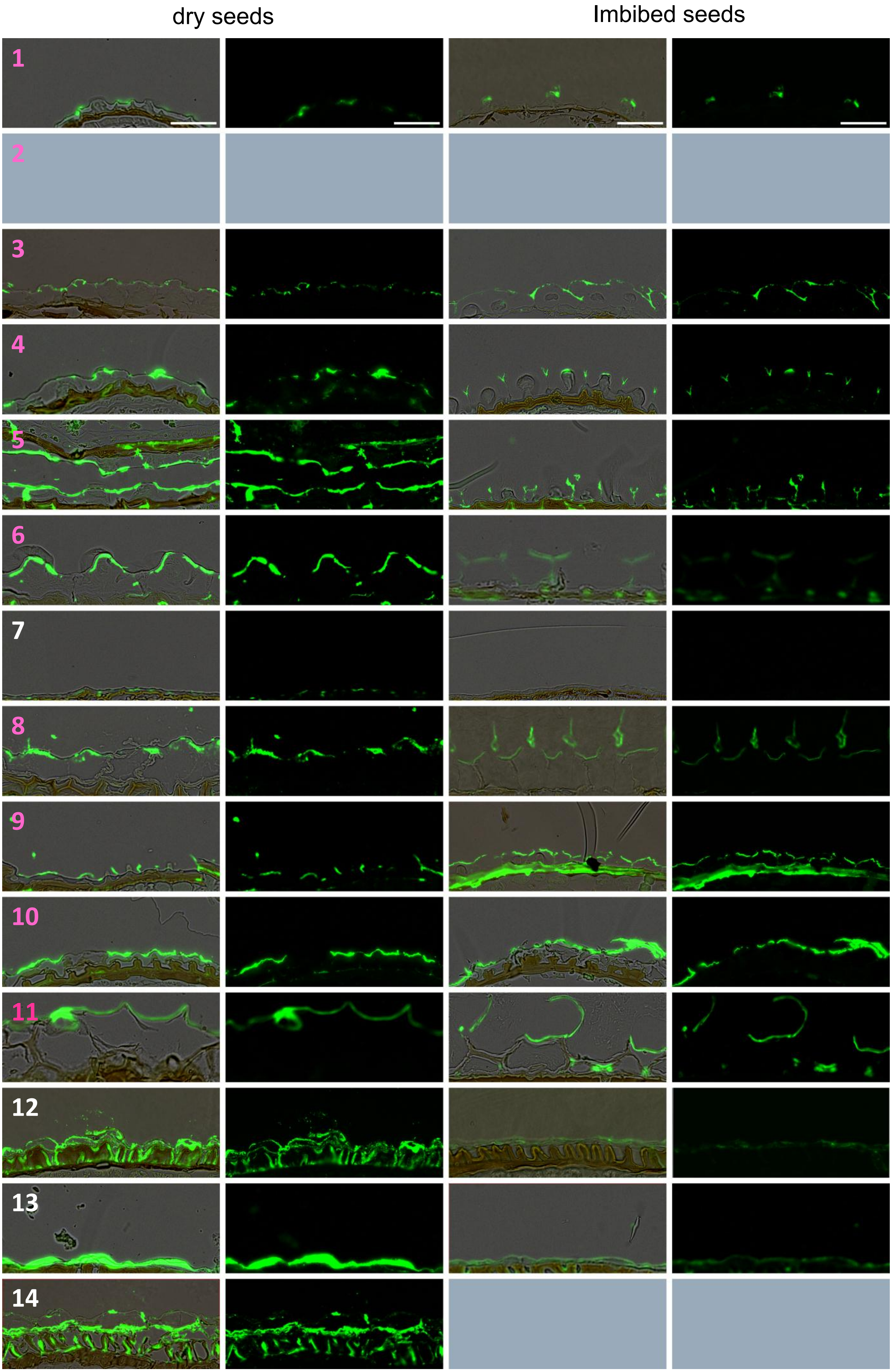

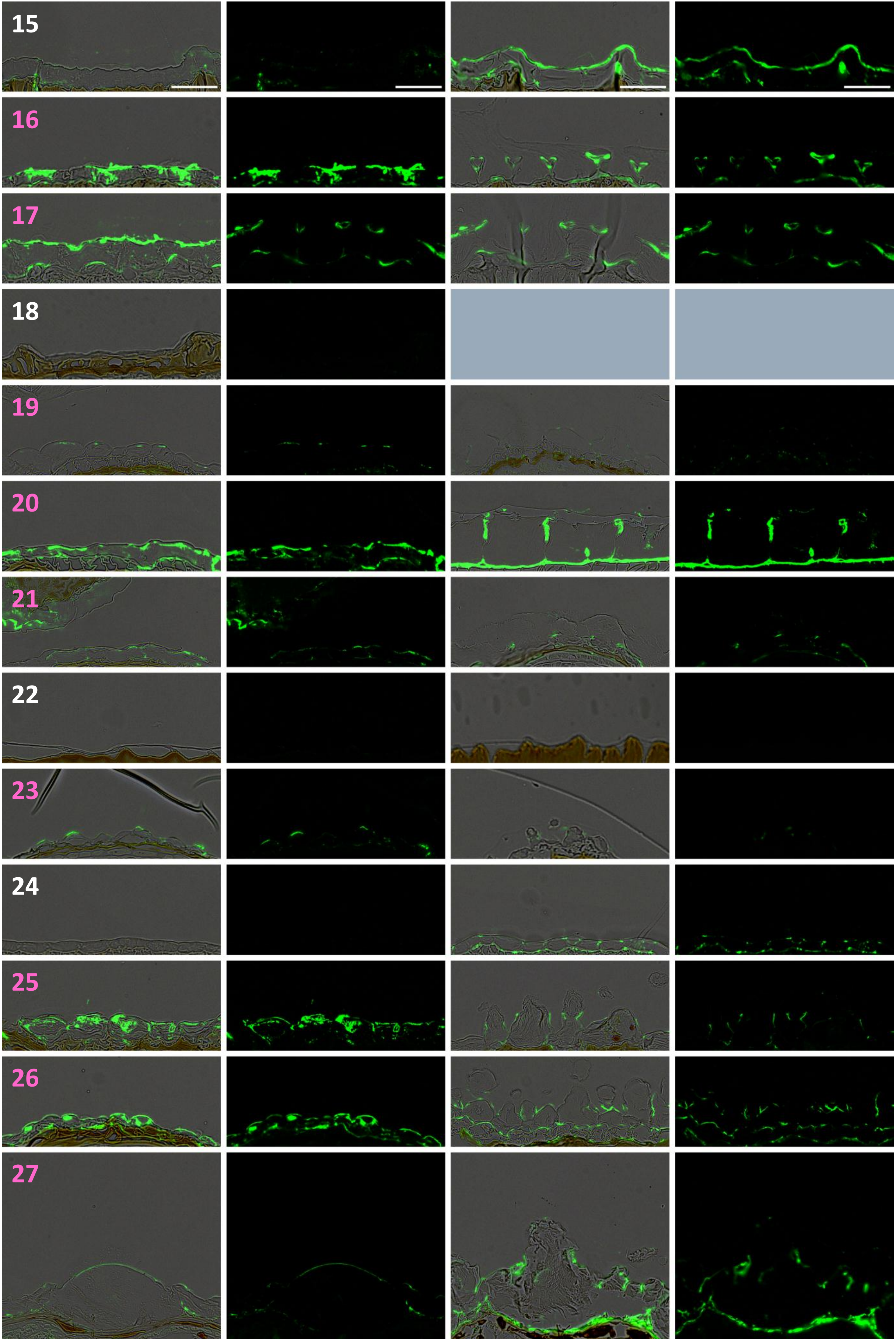

Figure S2: Immunofluorescence survey of the putative occurrence of a topochemical signature of the cell wall domain rupture zone in dry and imbibed seeds using the LM20 antibody. Serial sections of tissue arrays used for toluidine blue staining were used for immunofluorescence labeling with LM20 anti-partially demethylesterified homogalacturonan. This antibody has been previously shown to label the prefragilized cell wall microdomain that is ruptured upon *A. thaliana* seed imbibition (Francoz et al. 2019). Note the recurrent occurrence of punctuated labeling in the epidermal/MSC cell walls. Species numbers defined in Fig. **1** were colored in pink and white for myxospermous and non-myxospermous species, respectively. Grey panels correspond to unavailable or unexploitable samples. Bars: 50 µm.
